# Investigating the contribution of socio-economic position to ethnic inequalities in severe COVID-19 outcomes: population-based mediation analyses of national linked Scottish data

**DOI:** 10.1093/eurpub/ckaf078

**Published:** 2025-05-30

**Authors:** Sarah Amele, Eliud Kibuchi, Ronan McCabe, Evangelia Demou, Alastair H Leyland, Kirsten Hainey, Igor Rudan, Amanj Kurdi, Colin R Simpson, Lewis D Ritchie, Colin McCowan, Ting Shi, Patricia Irizar, Laia Becares, Aziz Sheikh, Anna Pearce, Srinivasa Vittal Katikireddi

**Affiliations:** MRC/CSO Social & Public Health Sciences Unit, University of Glasgow, Glasgow, United Kingdom; MRC/CSO Social & Public Health Sciences Unit, University of Glasgow, Glasgow, United Kingdom; MRC/CSO Social & Public Health Sciences Unit, University of Glasgow, Glasgow, United Kingdom; MRC/CSO Social & Public Health Sciences Unit, University of Glasgow, Glasgow, United Kingdom; MRC/CSO Social & Public Health Sciences Unit, University of Glasgow, Glasgow, United Kingdom; MRC/CSO Social & Public Health Sciences Unit, University of Glasgow, Glasgow, United Kingdom; Usher Institute, The University of Edinburgh, Edinburgh, United Kingdom; Strathclyde Institute of Pharmacy and Biomedical Sciences, Strathclyde University, Glasgow, United Kingdom; Department of Clinical Pharmacy, College of Pharmacy, Hawler Medical University, Kurdistan Regional Governorate, Erbil, Iraq; Department of Clinical Pharmacy, College of Pharmacy, Al-Kitab University, Kirkuk, Iraq; Department of Public Health Pharmacy and Management, School of Pharmacy, Sefako Makgatho Health Sciences University, Pretoria, South Africa; Usher Institute, The University of Edinburgh, Edinburgh, United Kingdom; School of Health, Wellington Faculty of Health, Victoria University of Wellington, Wellington, New Zealand; School of Medicine, Medical Sciences and Nutrition, University of Aberdeen, Aberdeen, United Kingdom; School of Medicine, University of St Andrews, St Andrews, United Kingdom; Usher Institute, The University of Edinburgh, Edinburgh, United Kingdom; Department of Sociology, School of Social Sciences, University of Manchester, Manchester, United Kingdom; Department of Global Health & Medicine, King's College London, London, United Kingdom; Usher Institute, The University of Edinburgh, Edinburgh, United Kingdom; MRC/CSO Social & Public Health Sciences Unit, University of Glasgow, Glasgow, United Kingdom; MRC/CSO Social & Public Health Sciences Unit, University of Glasgow, Glasgow, United Kingdom

## Abstract

We quantified the extent to which socio-economic position (SEP) contributed to ethnic inequalities in severe COVID-19 outcomes (hospitalization or death) in Scotland. We used linked 2011 Scottish Census and health records to assess whether ethnic inequalities were mediated by different SEP measures: area deprivation, educational status, household composition, and multigenerational household. We considered disaggregated ethnicities ‘White Scottish’, ‘White British or Irish’, ‘Other White’, ‘South Asian’, ‘African, Caribbean, or Black’, and ‘Other’. We applied marginal structural models to estimate causal pathways. Of the 3 297 205 individuals analysed, 38 213 (1.2%) had severe COVID-19 outcomes. South Asians had elevated risk of severe COVID-19 compared to White Scottish (hazard ratio: 1.7; 95% confidence interval: 1.5–1.9), while White British or Irish (hazard ratio: 0.7; confidence interval: 0.6–08) and other White (hazard ratio: 0.8; confidence interval: 0.7–0.9) had reduced risk. When holding area deprivation constant, the risk of severe COVID-19 declined by 16.5% for South Asians and 49.2% for White British or Irish; but increased for other White (75.4%). When holding education constant, the risk of severe COVID-19 reduced by 24.8% for White British or Irish and 20.6% for other White; but increased by 74.6% for South Asians. Only a slight change in risk was observed for the South Asians after holding household size and multigenerational household constant. Risk estimates for African, Caribbean or Black, and other groups were underpowered. SEP measures differed substantially in the extent to which they mediated ethnic inequalities in severe COVID-19. This highlights the necessity of addressing multiple dimensions of SEP that drive ethnic inequalities.

## Introduction

Minority ethnic groups worldwide faced a disproportionately high risk of COVID-19 [1]. Ethnic inequalities in severe COVID-19 (hospitalizations or deaths) were likely due to structural, institutional, and interpersonal racism [2], which generates and reinforces inequalities in access to health, social, and economic resources [2–4]. Studies have shown that socio-economic position (SEP), an aggregate concept including resource and prestige-based measures linked to an individual’s social class [5], significantly influenced differential exposure and vulnerability to severe acute respiratory syndrome coronavirus 2 (SARS-CoV-2) infection and severe COVID-19 outcomes [2]. However, it remains unclear whether the effects of ethnicity on severe COVID-19 in Scotland were mediated by SEP measures.

Ethnic inequalities in severe COVID-19 may arise from differential exposure to SARS-CoV-2 infection. Structural racism likely influences exposure through various SEP dimensions such as employment [6], education [7], and housing [8], leading to economic exclusion and health inequalities [9]. Institutional racism in sectors like education [7] and labour [6] exacerbates these inequalities through discrimination [9–12]. Minority ethnic groups are over-represented in deprived neighbourhoods [13], and in insecure employment and essential occupations like healthcare and transportation, where self-isolation was difficult during COVID-19 [14]. Essential workers had inadequate PPE access due to discrimination, increasing their exposure risk [15]. Additionally, the high burden of comorbidities and the likelihood of living in overcrowded [2] and multigenerational households [8] contributed to the increased risk of severe COVID-19 outcomes for minority ethnic groups.

No studies have investigated the causal pathways connecting SEP measures as mediators of the relationship between ethnicity and severe COVID-19 outcomes in Scotland. However, a UK Biobank study found that deprivation mediates the excess COVID-19 risk among minority ethnic groups in the UK [16]. One challenge in mediation analysis is handling intermediate confounders, which can lead to biased estimates if not properly accounted for [17]. Traditional multivariable regression can lead to bias results in the presence of exposure-induced mediator–outcome confounders, making traditional methods unsuitable for mediation analysis. Therefore, we used marginal structural models (MSMs) to address this [18]. MSMs were used to compute risk differences in severe COVID-19 outcomes across ethnic groups in Scotland and assess whether ethnic inequalities were mediated by SEP measures: area deprivation, educational status, household size, and multigenerational households. MSM handles potential confounding through weighting, allowing for unbiased estimation of SEP measures’ contribution to ethnic inequalities in COVID-19 outcomes [18]. This approach highlights the relevance of SEP dimensions in targeting policies to address structural racism and improve outcomes for minority ethnic individuals in future pandemic preparedness [18].

## Methods

### Study population and data

We used primary care data from the Early Assessment of Vaccine and Anti-Viral Effectiveness (EAVE II) study [19] linked to the 2011 Scottish Census; which is the latest census year available at time of analysis and forms the population spine for our cohort [20]. We included all individuals who were ≥16 years old, alive, and living in Scotland on 1 March 2020, the date of the first confirmed SARS-CoV-2 infection in Scotland. Individuals were followed until 17 April 2022, when free lateral flow testing of SARS-CoV-2 infection ended in Scotland.

Data from EAVE-II include primary care, testing, vaccination, hospitalization, and mortality data for approximately 99% of the Scottish population [19]. The Community Health Index (CHI) number, a unique numerical identifier used for all healthcare interactions in Scotland, was used to link the data. Hospitalization data were taken from the Scottish Morbidity Record, and mortality data from the National Records of Scotland deaths dataset. A detailed description of the dataset can be found in [20].

### Ethics

The Public Benefit and Privacy Panel Committees of Public Health Scotland and Scottish Government approved the linkage and analysis of the de-identified datasets for this project (2021–0115).

### Outcome

The primary outcome was severe COVID-19 (hospitalization or death). A COVID-19 hospitalization was defined as a hospitalization where a COVID-19 International Classification of Diseases (ICD-10) code (U07.1, U07.2) was listed in any diagnostic position or if the individual had a positive reverse transcription polymerase chain reaction (RT-PCR) test for SARS-CoV-2 in the 28 days prior to admission. A COVID-19 death was determined based on a COVID-19 ICD-10 code as the primary or secondary cause of death or a positive SARS-CoV-2 RT-PCR test in the 28 days prior to death.

### Ethnicity

Self-reported ethnicity was defined based on the 2011 Scottish Census, which included 16 ethnic groups. However, statistical power was low in several groups, so we aggregated the ethnicity variable into six categories [White Scottish (reference group), White British or Irish, other White, South Asian, African, Caribbean or Black, and other Ethnicity] to provide sufficient statistical power for our analyses. We also carried out sensitivity analysis using a binary variable [White (reference group) vs non-White], which may provide insight about potentially shared experiences across ethnic minority groups. See Supplementary Table S1 for full details on how the census ethnicity variable was aggregated. Ethnicity data from EAVE II were not used due to incompleteness and unreliableness [21].

### Mediators

We considered four different SEP mediators: (i) area deprivation, (ii) educational status, (iii) household size, and (iv) multigenerational households, all of which are implicated as risk factors for poor health outcomes across minority ethnic groups [22].

The Scottish Index of Multiple Deprivation (SIMD) measures relative area deprivation across datazones using seven weighted domains: employment, health, housing, income, education, access to services, and crime [23]. We used the 2020 SIMD obtained from the CHI register, defined using quintiles (1 = most deprived; 5 = least deprived) [23].

Education data from the 2011 census were categorized into degree (vocational or higher educational qualifications) and no degree (none, beyond secondary school). The education analysis only included individuals over 30 years, ensuring they were old enough to have completed higher education in 2011.

Household size was based on the 2011 Scottish Census and categorized into five groups: single occupancy, two-person (reference group), three-person, four-person, and five or more person households. Multigenerational households (grouped as yes or no) were defined as those where someone 65+ (1 March 2020) lived with someone at least 20 years younger.

### Confounders

**Figure 1.**
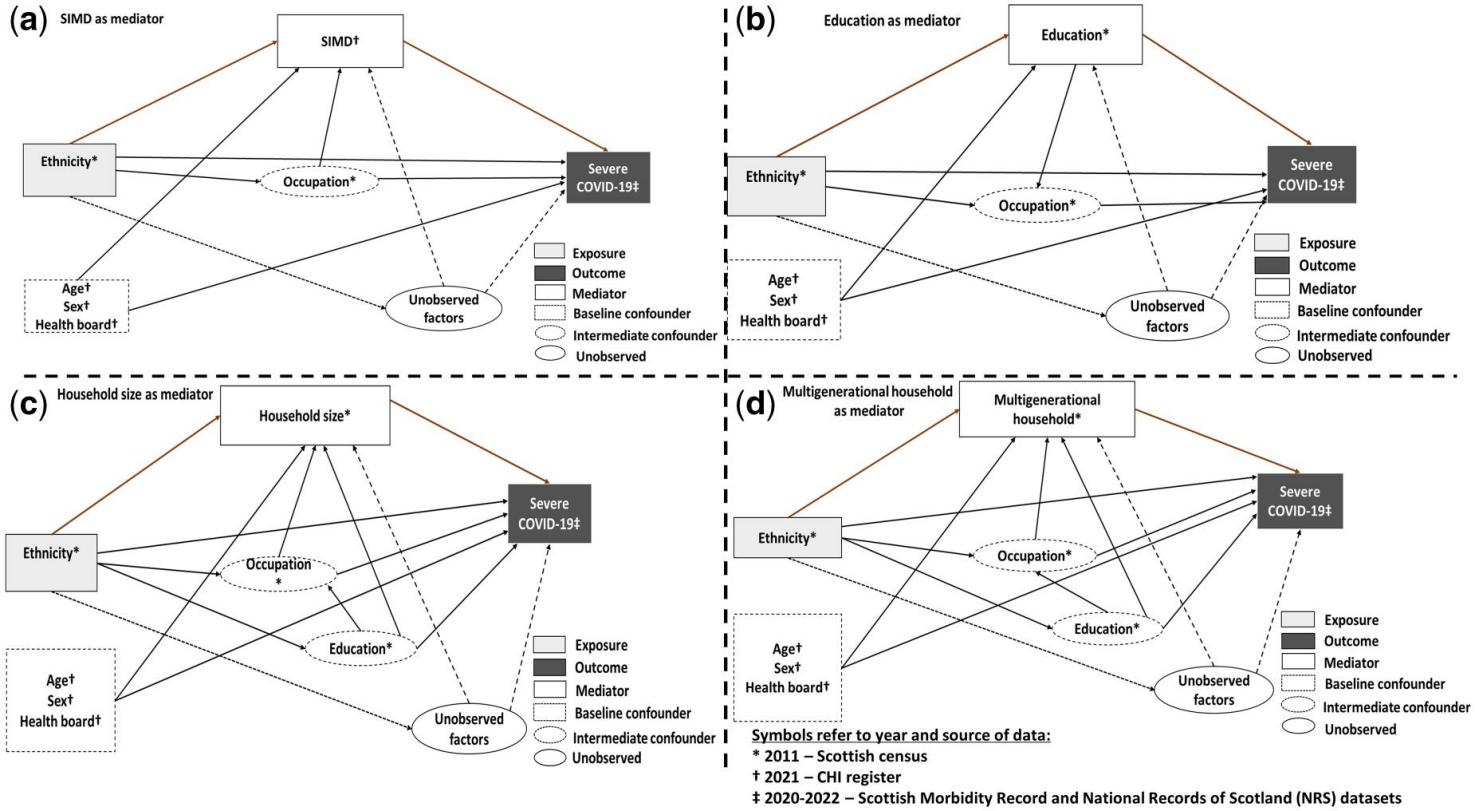
Directed acyclic graphs summarizing the relationship between ethnicity, SEPs mediators [SIMD (a), education (b), household size (c), and multigenerational household (d)], confounders, and COVID-19 outcomes.

We included three covariates in our analyses: age (continuous, specified with four-knot cubic splines), sex (binary), and health board (14 regional authorities responsible for delivering health services in Scotland). These were considered compositional variables that vary by ethnicity and affect COVID-19 outcomes but do not lie on their causal pathway [24]. Data on age, sex, and health board were taken from the CHI register and defined as of 1 March 2020. Intermediate confounders (confounders of the mediator–outcome relationship affected by ethnicity and therefore on the causal pathway with the COVID-19 outcome) differed for each of the four mediation analyses, as shown in the Direct Acyclic Graphs in Fig. 1.

Occupational risk was included as an intermediate confounder in all analyses. Education was an intermediate confounder for household size and multigenerational households but excluded for SIMD since it is used for its derivation.

Occupational risk, determined from the 2011 Scottish Census, was categorized into low, medium, high risk, and economically inactive, based on potential SARS-CoV-2 exposure. This classification was informed by prior assessments of infection and mortality risks from COVID-19 [24] and was independently conducted by five authors (E.K., R.M., A.P., E.D., and S.V.K.) using Standardized Occupational Classification codes. In cases of disagreement, authors discussed and reached a consensus on the appropriate risk level, as shown Supplementary Table S2. Except for S.V.K., the authors do not have clinical settings expertise.

### Statistical analysis

Ethnic differences in baseline characteristics of individuals included in this analysis were described using summary statistics.

To explore the extent to which inequalities could be addressed by adjusting SEP measures on the association between ethnicity and severe COVID-19, we used MSMs [18]. MSM employs inverse probability weights (IPWs) to consistently estimate causal effects from observational data [18]. In this study, we estimated both the total effect (TE) and the counterfactual disparity measure (CDM) of ethnicity on severe COVID-19 outcomes [18]. In our context, CDM represents the effect of ethnicity on the COVID-19 outcome that is not mediated by a given SEP mediator. Essentially, CDM helps identify how ethnic inequalities could be causally altered by changing SEP mediators. To account for intermediate confounding, we used MSM to create two sets of IPWs [25].

First, weights for ethnicity (exposure) were calculated to adjust for and accounted for sex, age, and health board. Second, mediator weights were computed to control for confounders of the mediator/outcome relationship (see Fig. 1). Weights for binary ethnicity (White vs non-White) were estimated using logistic regression models, while multinomial logistic models were used for multicategory ethnicity variables [18]. We stabilized weights by including the marginal probability of the observed exposure in the numerator. To maintain weights near a mean of 1, the top and bottom 1% were truncated to avoid extremes. The exposure and mediator weights were then combined into one weight for the CDM models (TE models only included the exposure weight).

The TE of ethnicity on severe COVID-19 was estimated using a Cox proportional hazards model, with baseline covariates included for double robustness. The controlled direct effect (CDE) of ethnicity on severe COVID-19 was then estimated by adjusting for the mediator of interest, allowing for an interaction between the exposure and mediator, and the baseline covariates.

Finally, we calculated the proportion change (PC), this is the proportion of ethnic inequalities in severe COVID-19 outcomes that would be eliminated if the mediator was held at a certain value [26]. For relative measures of effect (such as hazard ratios), PC is calculated as shown in Equation (1):


(1) $$\mathrm{PC}=\frac{\left(\mathrm{TE}-\mathrm{CDM}\right)}{\left(\mathrm{TE}-1\right)}$$

We used variance-covariance matrices to derive standard errors and confidence intervals of the fitted model coefficients (TE and CDE), which enabled computation of 95% confidence intervals (CIs) for PC. All analysis was completed in R (version 4.2.0) in a Trusted Research Environment.

## Results

There were 5 121 530 individuals included in the EAVE-II dataset, 51 138 of whom were missing data on their health board, 133 955 missing ethnicity data, and 433 632 missing education data. Table 1 shows the baseline characteristics of the remaining 3 297 205 individuals aged 16+ included in this analysis, categorized by ethnic group. The study population was predominantly White Scottish (86.0%), followed by White British or Irish (8.8%), other White (2.5%), South Asian (1.2%), other (1.1%), and African, Caribbean, or Black (0.4%). There were more females (52.4%) than males (47.6%). Minority ethnic groups and a higher proportion of younger individuals, while older individuals were more common among White Scottish and White British or Irish, with older individuals more prevalent in the latter groups. The highest proportion of individuals experiencing severe COVID-19 was among South Asians (1.3%), followed by White Scottish (1.2%), White British or Irish (0.8%), African, Caribbean, or Black (0.7%), other (0.6%), and other White (0.5%).

**Table 1.** Socio-demographic characteristics by ethnicity, for all individuals aged 16+ (frequency, %)

–		Overall	White Scottish	White British or Irish	Other White	South Asian	African, Caribbean or Black	Other
Overall		3 297 205, 100.0	2 837 058, 86.0	289 298, 8.8	82 786, 2.5	40 499, 1.2	12 470, 0.4	35 094, 1.1
Age	16–20	75, 0.0	–	–	–	–	–	–
	21–25	30 406, 0.9	–	–	–	–	–	–
	26–30	256 304, 7.8	228 957, 8.1	13 574, 4.7	5063, 6.1	3995, 9.9	1063, 8.5	3652, 10.4
	31–35	256 933, 7.8	219 492, 7.7	16 106, 5.6	10 511, 12.7	4930, 12.2	1249, 10.0	4645, 13.2
	36–40	263 663, 8.0	213 936, 7.5	19 263, 6.7	17 575, 21.2	6163, 15.2	2074, 16.6	4652, 13.3
	41–45	253 361, 7.7	204 236, 7.2	22 221, 7.7	13 887, 16.8	5843, 14.4	2448, 19.6	4726, 13.5
	46–50	300 291, 9.1	250 483, 8.8	28 950, 10.0	9653, 11.7	4873, 12.0	1866, 15.0	4466, 12.7
	51–55	343 298, 10.4	294 847, 10.4	32 522, 11.2	7111, 8.6	3955, 9.8	1347, 10.8	3516, 10.0
	56–60	343 782, 10.4	298 669, 10.5	32 727, 11.3	5594, 6.8	2952, 7.3	935, 7.5	2905, 8.3
	61–65	310 349, 9.4	271 549, 9.6	28 923, 10.0	4446, 5.4	2612, 6.4	543, 4.4	2276, 6.5
	>65	938 743, 28.5	827 387, 29.2	93 572, 32.3	8493, 10.3	4614, 11.4	827, 6.6	3850, 11.0
Sex	Male	1 567 877, 47.6	1 343 875, 47.4	141 895, 49.0	37 812, 45.7	21 168, 52.3	6463, 51.8	16 664, 47.5
	Female	1 729 328, 52.4	1 493 183, 52.6	147 403, 51.0	44 974, 54.3	19 331, 47.7	6007, 48.2	18 430, 52.5
Health board	Ayrshire and Arran	244 281, 7.4	224 502, 7.9	15 113, 5.2	2332, 2.8	831, 2.1	250, 2.0	1253, 3.6
	Borders	70 278, 2.1	56 301, 2.0	11 740, 4.1	1667, 2.0	149, 0.4	91, 0.7	330, 0.9
	Dumfries and Galloway	97 297, 3.0	78 951, 2.8	16 132, 5.6	1334, 1.6	267, 0.7	132, 1.1	481, 1.4
	Forth Valley	178 415, 5.4	159 108, 5.6	13 603, 4.7	3142, 3.8	1280, 3.2	288, 2.3	994, 2.8
	Grampian	362 324, 11.0	298 710, 10.5	41 718, 14.4	13 047, 15.8	2622, 6.5	2117, 17.0	4110, 11.7
	Highland	194 322, 5.9	156 048, 5.5	30 594, 10.6	5517, 6.7	603, 1.5	304, 2.4	1256, 3.6
	Lothian	508 361, 15.4	411 005, 14.5	55 607, 19.2	23 176, 28.0	7423, 18.3	2728, 21.9	8422, 24.0
	Orkney	13 516, 0.4	–	–	–	–	–	–
	Shetland	14 217, 0.4	–	–	–	–	–	–
	Western Isles	16 678, 0.5	14 536, 0.5	1791, 0.6	213, 0.3	53, 0.1	14, 0.1	71, 0.2
	Fife	224 661, 6.8	196 946, 6.9	19 874, 6.9	4294, 5.2	1481, 3.7	464, 3.7	1602, 4.6
	Tayside	251 769, 7.6	217 573, 7.7	22 232, 7.7	6719, 8.1	2345, 5.8	625, 5.0	2275, 6.5
	Greater Glasgow and Clyde	712 529, 21.6	621 870, 21.9	38 797, 13.4	15 781, 19.1	19 550, 48.3	4699, 37.7	11 832, 33.7
	Lanarkshire	408 557, 12.4	378 952, 13.4	17 634, 6.1	5098, 6.2	3842, 9.5	732, 5.9	2299, 6.6
Occupation	Low risk	1 346 460, 40.8	1 167 343, 41.1	128 668, 44.5	28 210, 34.1	9706, 24.0	3651, 29.3	8882, 25.3
	Med risk	1 183 541, 35.9	1 025 738, 36.2	85 904, 29.7	37 569, 45.4	16 572, 40.9	3755, 30.1	14 003, 39.9
	High risk	583 180, 17.7	490 649, 17.3	65 479, 22.6	12 083, 14.6	5748, 14.2	3275, 26.3	5946, 16.9
	Economically inactive	184 024, 5.6	153 328, 5.4	9247, 3.2	4924, 5.9	8473, 20.9	1789, 14.3	6263, 17.8
SIMD	1 (most deprived)	628 290, 19.1	565 005, 19.9	26 896, 9.3	18 932, 22.9	6463, 16.0	4446, 35.7	6548, 18.7
	2	662 411, 20.1	588 139, 20.7	41 333, 14.3	17 147, 20.7	7417, 18.3	2414, 19.4	5961, 17.0
	3	672 916, 20.4	573 360, 20.2	70 027, 24.2	15 202, 18.4	6703, 16.6	1790, 14.4	5834, 16.6
	4	676 666, 20.5	569 065, 20.1	74 514, 25.8	14 953, 18.1	9248, 22.8	1826, 14.6	7060, 20.1
	5 (least deprived)	656 922, 19.9	541 489, 19.1	76 528, 26.5	16 552, 20.0	10 668, 26.3	1994, 16.0	9691, 27.6
Number of people in household	1	504 093, 15.3	447 373, 15.8	40 944, 14.2	8139, 9.8	2198, 5.4	1753, 14.1	3686, 10.5
	2	1 159 389, 35.2	997 183, 35.1	117 103, 40.5	27 118, 32.8	5859, 14.5	2952, 23.7	9174, 26.1
	3	699 287, 21.2	607 871, 21.4	53 562, 18.5	20 470, 24.7	6674, 16.5	2695, 21.6	8015, 22.8
	4	622 083, 18.9	536 984, 18.9	50 039, 17.3	16 300, 19.7	8658, 21.4	2539, 20.4	7563, 21.6
	5+	312 353, 9.5	247 647, 8.7	27 650, 9.6	10 759, 13.0	17 110, 42.2	2531, 20.3	6656, 19.0
Education	Degree	871 802, 26.4	658 276, 23.2	132 928, 45.9	41 782, 50.5	16 307, 40.3	6429, 51.6	16 080, 45.8
	No degree	2 425 403, 73.6	2 178 782, 76.8	156 370, 54.1	41 004, 49.5	24 192, 59.7	6041, 48.4	19 014, 54.2
Multigenerational household	No	3 164 068, 96.0	2 722 668, 96.0	278 763, 96.4	80 892, 97.7	36 135, 89.2	12 181, 97.7	33 429, 95.3
	Yes	133 137, 4.0	114 390, 4.0	10 535, 3.6	1894, 2.3	4364, 10.8	289, 2.3	1665, 4.7
Severe COVID-19 (i.e. hospitalization or death)	No	3 258 992, 98.8	2 802 443, 98.8	286 953, 99.2	82 370, 99.5	39 969, 98.7	12 383, 99.3	34 874, 99.4
	Yes	38 213, 1.2	34 615, 1.2	2345, 0.8	416, 0.5	530, 1.3	87, 0.7	220, 0.6

Only 9.3% of the White British or Irish group were in the most deprived SIMD quintile, while 35.7% of African, Caribbean, or Black individuals were in this quintile. The White British or Irish, South Asian, and other ethnic groups had a higher proportion of individuals in the least deprived SIMD quintile (26.5%, 26.3%, and 27.6%, respectively). Few South Asian individuals lived in single-occupancy households (5.4%) compared to all other ethnic groups (>10%) and were more likely to live in households with five or more individuals (42.2%) than African, Caribbean, or Black (20.3%), Other ethnic groups (19.0%), and White Scottish, White British or Irish, and other White individuals (∼10%). The distribution of baseline characteristics for individuals aged 30+ (working age during the 2011 census) and 30–64 years (working age in 2020) are in Supplementary Tables S3 and S4, respectively.

Table 2 presents TE and CDM of disaggregated ethnic groups on severe COVID-19 with corresponding summary statistics of IPWs presented in Supplementary Table S5. South Asian individuals had a higher risk of severe COVID-19 compared to White Scottish individuals [TE hazards ratio (HR): 1.7; 95% CI: 1.5, 1.9]. White British or Irish and other White individuals had a lower hazard of severe COVID-19 compared to White Scottish individuals (TE HR: 0.7; CI: 0.6–0.8 and TE HR: 0.8; CI: 0.7–0.9, respectively). Estimates for African, Caribbean, or Black and other ethnic groups were imprecise due to the small number of events.

**Table 2.** TE and CDMs hazard ratios (HR) and PC in severe COVID-19 on disaggregated ethnicity

		HR (95% CI)	PC (95% CI)
TE			
Ethnicity	White Scottish	1	N/A
	White British or Irish	0.71 (0.68–0.75)	N/A
	Other White	0.80(0.72–0.89)	N/A
	South Asian	1.69 (1.53–1.86)	N/A
	African, Caribbean or Black	1.07 (0.83–1.38)	N/A
	Other	0.92 (0.79–1.07)	N/A
CDMs, after accounting for:			
SIMD (five groups)	White Scottish	1	
	White British or Irish	0.85 (0.78–0.94)	49.20% (48.24%–50.22%)
	Other White	0.67 (0.54–0.82)	−75.35% (−79.43 to −71.26%)
	South Asian	1.55 (1.2–2.01)	16.50% (14.28%–18.73%)
	African, Caribbean or Black	0.82 (0.55–1.24)	^a^
	Other	1.05 (0.78–1.41)	^a^
Education (binary—population aged 30+)	White Scottish	1	
	White British or Irish	0.78 (0.72–0.86)	24.8% (24.03%–25.65%)
	Other White	0.85 (0.7–1.02)	20.59% (17.48%–23.70%)
	South Asian	2.08 (1.72–2.52)	−74.62% (−77.09% to −72.14%)
	African, Caribbean or Black	1.73 (1.22–2.47)	^a^
	Other	1.36 (1.07–1.73)	^a^
Household size (five groups)	White Scottish	1	
	White British or Irish	0.70 (0.66–0.76)	−4.86% (−5.51% to −4.21%)
	Other White	0.81 (0.68–0.97)	−4.47% (−7.61% to −1.32%)
	South Asian	1.55 (1.19–2.01)	9.81% (7.54%–12.08%)
	African, Caribbean or Black	0.54 (0.3–0.97)	^a^
	Other	0.78 (0.59–1.03)	^a^
Multigenerational household (binary)	White Scottish	1	
	White British or Irish	0.70 (0.67–0.74)	−3.59% (−4.10% to −3.08%)
	Other White	0.80 (0.72–0.90)	−1.09% (−8.37% to 6.20%)
	South Asian	1.72 (1.54–1.92)	−7.79% (−9.02% to −6.57%)
	African, Caribbean or Black	1.13 (0.87–1.46)	^a^
	Other	0.95 (0.81–1.11)	^a^

a Excluded due to low numbers leading to imprecise estimates.

When holding SIMD constant, South Asians had a higher hazard of severe COVID-19 compared to White Scottish individuals (CDM: 1.5 (1.2, 2.0)). The lower risk for White British (0.8 (0.7, 0.9)) and Other White (0.6 (0.5, 0.8)) individuals decreased further compared to White Scottish individuals. Severe COVID-19 risk increased among South Asians (2.1 (1.7, 2.5)), African, Caribbean, or Black (1.7 (1.2, 2.5)), and other ethnic groups (1.4 (1.1, 1.7)) after adjusting for education. When household size differences were held constant, White British (0.7 (0.6, 0.8)), other White (0.8 (0.6, 1.0)), and African, Caribbean, or Black groups (0.5 (0.1, 1.0)) had a lower hazard of severe COVID-19 compared to the White Scottish group. However, the CDM for South Asians remained higher (1.6 (1.2, 2.0)) compared to White Scottish individuals after holding household size constant. When multigenerational household differences were held constant, the CDM for severe COVID-19 was 0.7 (0.6, 0.8) for White British, 0.8 (0.7, 0.9) for other White, and 1.7 (1.5, 1.9) for South Asians compared to White Scottish individuals. There were no significant differences in CDM between African, Caribbean, or Black, and other ethnic groups compared to the White Scottish groups after adjusting for multigenerational households.

In relative terms, the elevated risk for South Asian individuals reduced by 16.5% (95% CI: 14.3%, 18.7%) and 9.5% (7.5%, 12.1%) if inequalities in SIMD and household size were held constant, respectively (see PC estimates in Table 2). The estimated reduction in ethnic inequalities was 24.8% (24.0%, 25.6%) for White British or Irish individuals and 20.6% (17.5%, 23.7%) for Other White individuals if education differences were held constant. However, inequalities for South Asians increased by 74.6% if education differences were held constant. The estimated increase in inequalities was 4.9% (−5.5%, −4.2%) for White British or Irish individuals and −4.5% (−7.6%, −1.3%) for other White individuals if household size differences were held constant. However, holding household size differences constant led to an estimated increase in severe COVID-19 risk of 9.8% (7.5%, 12.1%) for South Asians. If multigenerational household differences were held constant, ethnic inequalities in severe COVID-19 would increase by 3.6% (−4.1%, −3.1%) for White British individuals, 1.1% (−8.4%, −6.2%) for other White individuals, and 7.8% (−9.0%, −6.6%) for South Asians. PC estimates for the African, Caribbean, or Black and other ethnic groups were not reported due to their low sample sizes.

The TE and CDM estimates comparing non-White ethnic groups to the White ethnic group and summary statistics for the IPWs are presented in Supplementary Tables S6 and S7, respectively. Overall, non-White individuals had a higher risk of severe COVID-19 compared to White individuals (TE: 1.4 (1.3, 1.5)). When SIMD and household size differences were held constant, the risk of severe COVID-19 for the non-White group reduced by 52.7% and 88.6%, respectively. However, it increased by 98.8% and 3.2% if education and multigenerational household differences were held constant, respectively.

## Discussion

We found that South Asians had a higher risk of severe COVID-19 compared to the White Scottish group, while White British or Irish and other White groups had a lower risk. There was no difference in severe COVID-19 risk between African, Caribbean, or Black, other groups, and White Scottish group, contradicting previous findings [27], likely due to different age groups in the datasets (+30 years in our study vs. +16 years in the previous study). The elevated risk of severe COVID-19 among South Asians would reduce if SIMD and household size differences were eliminated. However, if education and multigenerational household differences were constant, the risk would increase. For the White British or Irish group, the risk would decrease without SIMD and education differences. For the other White group, the risk would increase if SIMD and household size differences were constant and decrease without educational differences. For aggregated ethnic groups, the risk among the non-White group would reduce without SIMD and household size differences and increase without differences in education and multigenerational households.

These results show that patterns significantly differ based on the SEP mediator measures used to assess the effects of ethnicity on severe COVID-19 outcomes. This supports Fischbacher et al.’s finding that different SEP measures relate differently to ethnic groups in Scotland [22]. The higher education among South Asians compared to White Scottish may explain the increase in severe COVID-19 after holding education constant [22]. Though higher education generally improves health [28], it does not always lead to employment and income success for UK minority ethnic groups [29]. Occupational risks, such as limited protective gear for healthcare workers, also highlight institutional and structural inequalities that shaped ethnic inequalities during the pandemic [30]. However, we do not believe that this makes them invalid markers of SEP but instead consider them to shed light on different mechanisms through which ethnic inequalities may arise.

The decrease in severe COVID-19 among South Asians when SIMD and household size differences are removed may be explained by the high number of South Asians (except Indians) living in the most deprived neighbourhoods [31] and their larger household sizes compared to White Scottish individuals [32]. Living in multigenerational households increases social, psychological, and financial capital, which is associated with improved health [33]. This may have benefited South Asians, therefore, holding differences in multigenerational households constant removes these benefits, and increases the risk of severe COVID-19.

Our findings highlight the importance of using multiple SEP measures [22] when evaluating the mediating role of SEP on the impact of racism on health outcomes, as ethnicity captures how ethnic groups are racialized. Interventions should address SEP inequities faced by minority ethnic groups and target racism at institutional and interpersonal levels [34]. Additionally, it is crucial to consider how minority ethnic groups are racialized differently and the impact of other factors, such as timing and reasons for migration, on their SEP [35]. Ethnic inequalities in disease exposure and vulnerability are embedded in multiple structures that reinforce inequitable laws, policies, and the unequal distribution of resources [9–12], as highlighted in the Priority Public Health Conditions framework [2].

A major strength of this analysis was the use of self-defined ethnicity data from the Scottish Census, considered the ‘gold-standard’ of ethnicity data in Scotland [21]. Additionally, using MSM provided less biased estimates of the relative contribution of different SEP measures to ethnic inequalities in COVID-19 outcomes, enabling comparison of how these measures differentially mediate the effect of ethnicity on severe COVID-19 outcomes [18]. This is crucial when evaluating the effectiveness of various public health policies.

Our analysis also has some limitations. Firstly, we used the 2011 Scottish Census, which was almost 10 years old during linkage, as the 2022 Scottish Census was unavailable. While ethnic identity can change over time (4% changed between 2001–11, mainly ‘other’ and ‘mixed’ categories), major ethnic groups remained stable, minimizing bias. Multigenerational household and education data may have changed, particularly for younger individuals, leading to possible misclassification. Moreover, migration may have altered the adult population’s ethnic composition, meaning the results may not fully reflect the current distribution. Although some cases of severe COVID-19 may have been misdiagnosed, introducing measurement error into the outcome, it is unlikely to result in substantial bias to our estimates of mediation of ethnic inequalities as we would not anticipate differential misclassification across ethnic groups. Secondly, SIMD 2020 is an area-based measure, assuming everyone a given area experiences the same deprivation level, potentially masking individual variations. Third, due to the small number of severe COVID-19-related outcomes, ethnicity was aggregated into binary and categorical variables. Findings may differ due to heterogeneity within groups, particularly African, Caribbean, or Black, and other ethnicities, which had small sample sizes [21, 27].

Fourth, there is potential for residual confounding in TE and CDM estimates from incorrectly specified IPW models [18]. However, this is more pertinent with continuous variables, and both the exposure and mediators in this analysis were binary/categorical [18]. Fifth, MSM assumes no unmeasured mediator–outcome confounding, which is unlikely. Although linking census and health record data maximized the variables we could adjust for, unmeasured confounding, such as structural racism, may still have occurred, which future studies should aim to explore. Lastly, the relationship between ethnicity, SEP measures, and health inequalities in Scotland may differ from other countries, such as the United States, where SEP and ethnicity are more strongly correlated [36]. Caution should be exercised when generalizing these findings to other settings.

In conclusion, SEP measures substantially differed in how they mediated ethnic inequalities in severe COVID-19 outcomes in Scotland. While future infectious diseases pandemics are a likely inevitability, it is important to minimize the disproportionate impacts on marginalized groups. Our findings highlight the need for comprehensive, inclusive, and targeted measures to address the multiple dimensions of SEP that drive ethnic inequalities in health outcomes, to ensure they are not exacerbated by future pandemics. Future studies should investigate SEP measures not considered here and explore how racism drives ethnic inequalities in SEP.

## Supplementary Material

ckaf078_Supplementary_Data

## Data Availability

The data used in this study include sensitive category individual-level data. To prevent disclosure, these data are not publicly available but are available for research purposes through successful application to NHS Scotland Public Benefit and Privacy Panel for Health and Social Care (HSC-PBPP) and National Records of Scotland (NRS). Researchers interested in the data may contact the Electronic Data Research and Innovation Service (eDRIS) phs.edris@phs.scot.
