# Supplementary material for: Investigating the contribution of socio-economic position to ethnic inequalities in severe COVID-19 outcomes: population-based mediation analyses of national linked Scottish data
Source: Eur J Public Health. 2025 May 30;35(4):788–94. doi: 10.1093/eurpub/ckaf078 (PMC12311338; doi:10.1093/eurpub/ckaf078)
Supplement: ckaf078_Supplementary_Data [file ckaf078_supplementary_data.docx]

**Supplementary data: Investigating the contribution of socioeconomic position to ethnic inequalities in severe COVID-19 outcomes: Population-based mediation analyses of national linked Scottish data**

Table S1**:** Ethnicity classifications based in 2011 Scottish Census

| ***Dis-aggregated ethnic groups in 2011 Scottish Census*** | ***Categorical ethnicity variable*** | ***Binary ethnicity variable*** |
| --- | --- | --- |
| White Scottish | White Scottish | White |
| White Other British | White Other British or Irish |  |
| White Irish |  |  |
| 0White Gypsy/Traveller | Other White |  |
| White Polish |  |  |
| Other White |  |  |
| Pakistani, Pakistani Scottish or Pakistani British (referred to in main text as Pakistani) | South Asian | Non-White |
| Indian, Indian Scottish, or Indian British (referred to in main text as Indian) |  |  |
| Bangladeshi, Bangladeshi Scottish or Bangladeshi British (referred to in main text as Bangladeshi) |  |  |
| African, African Scottish, or African British (referred to in main text as African) | African, Caribbean, or Black |  |
| Caribbean, Caribbean Scottish or Caribbean British/Black, Black Scottish or Black British (referred to in main text as African or Black) |  |  |
| Arab, Arab Scottish, or Arab British (referred to in main text as Arab) | Other Ethnicity |  |
| Chinese, Chinese Scottish or Chinese British (referred to in main text as Chinese) |  |  |
| Other Asian |  |  |
| Mixed or Multiple Ethnicity |  |  |
| Other Ethnicity |  |  |

Table S2: List of occupations categorised into different COVID-19 risk levels.

| **3-digit SOC group** | **SOC Number** | **COVID-19 occupation risk** |
| --- | --- | --- |
| 111 111 'Chief Executives and Senior Officials' | 111 | Low |
| 112 112 'Production Managers and Directors' | 112 | Low |
| 113 113 'Functional Managers and Directors' | 113 | Low |
| 115 115 'Financial Institution Managers and Directors' | 115 | Low |
| 116 116 'Managers and Directors in Transport and Logistics' | 116 | Medium |
| 117 117 'Senior Officers in Protective Services' | 117 | Medium |
| 118 118 'Health and Social Services Managers and Directors' | 118 | Medium |
| 119 119 'Managers and Directors in Retail and Wholesale' | 119 | Medium |
| 121 121 'Managers and Proprietors in Agriculture Related Srvcs' | 121 | Low |
| 122 122 'Managers and Proprietors in Hsptlty and Leisure Srvcs' | 122 | Medium |
| 124 124 'Managers and Proprietors in Health and Care Services' | 124 | Medium |
| 125 125 'Managers and Proprietors in Other Services' | 125 | Medium |
| 211 211 'Natural and Social Science Professionals' | 211 | Low |
| 212 212 'Engineering Professionals' | 212 | Low |
| 213 213 'IT and Telecommunications Professionals' | 213 | Low |
| 214 214 'Conservation and Environment Professionals' | 214 | Low |
| 215 215 'Research and Development Managers ' | 215 | Low |
| 221 221 'Health Professionals' | 221 | High |
| 222 222 'Therapy Professionals' | 222 | High |
| 223 223 'Nursing and Midwifery Professionals' | 223 | High |
| 231 231 'Teaching and Educational Professionals' | 231 | High |
| 241 241 'Legal Professionals' | 241 | Low |
| 242 242 'Business, Research and Administrative Professionals' | 242 | Low |
| 243 243 'Architects, Town Planners and Surveyors' | 243 | Low |
| 244 244 'Welfare Professionals' | 244 | High |
| 245 245 'Librarians and Related Professionals' | 245 | Low |
| 246 246 'Quality and Regulatory Professionals' | 246 | Low |
| 247 247 'Media Professionals' | 247 | Low |
| 311 311 'Science, Engineering and Production Technicians' | 311 | Low |
| 312 312 'Draughtspersons and Related Architectural Technicians' | 312 | Low |
| 313 313 'Information Technology Technicians' | 313 | Low |
| 321 321 'Health Associate Professionals' | 321 | High |
| 323 323 'Welfare and Housing Associate Professionals' | 323 | High |
| 331 331 'Protective Service Occupations' | 331 | High |
| 341 341 'Artistic, Literary and Media Occupations' | 341 | Low |
| 342 342 'Design Occupations' | 342 | Low |
| 344 344 'Sports and Fitness Occupations' | 344 | Medium |
| 351 351 'Transport Associate Professionals' | 351 | Low |
| 352 352 'Legal Associate Professionals' | 352 | Low |
| 353 353 'Business, Finance and Related Associate Professionals' | 353 | Low |
| 354 354 'Sales, Marketing and Related Associate Professionals' | 354 | Low |
| 355 355 'Conservation and Environmental Assct Professionals' | 355 | Low |
| 356 356 'Public Services and Other Associate Professionals' | 356 | Low |
| 411 411 'Administrative Occupations: Gvrnmnt and Related Orgs' | 411 | Low |
| 412 412 'Administrative Occupations: Finance' | 412 | Low |
| 413 413 'Administrative Occupations: Records' | 413 | Low |
| 415 415 'Other Administrative Occupations' | 415 | Low |
| 416 416 'Administrative Occupations: Office Mngrs and Sprvsrs' | 416 | Low |
| 421 421 'Secretarial and Related Occupations' | 421 | Medium |
| 511 511 'Agricultural and Related Trades' | 511 | Low |
| 521 521 'Metal Forming, Welding and Related Trades' | 521 | Low |
| 522 522 'Metal Machining, Fitting and Instrument Making Trades' | 522 | Low |
| 523 523 'Vehicle Trades' | 523 | Low |
| 524 524 'Electrical and Electronic Trades' | 524 | Low |
| 525 525 'Skilled Metal, Electrical and Electronic Trds Sprvsrs' | 525 | Low |
| 531 531 'Construction and Building Trades' | 531 | Low |
| 532 532 'Building Finishing Trades' | 532 | Low |
| 533 533 'Construction and Building Trades Supervisors' | 533 | Low |
| 541 541 'Textiles and Garments Trades' | 541 | Low |
| 542 542 'Printing Trades' | 542 | Low |
| 543 543 'Food Preparation and Hospitality Trades' | 543 | Medium |
| 544 544 'Other Skilled Trades' | 544 | Low |
| 612 612 'Childcare and Related Personal Services' | 612 | High |
| 613 613 'Animal Care and Control Services' | 613 | Low |
| 614 614 'Caring Personal Services' | 614 | High |
| 621 621 'Leisure and Travel Services' | 621 | Medium |
| 622 622 'Hairdressers and Related Services' | 622 | Medium |
| 623 623 'Housekeeping and Related Services' | 623 | Medium |
| 624 624 'Cleaning and Housekeeping Managers and Supervisors' | 624 | Medium |
| 711 711 'Sales Assistants and Retail Cashiers' | 711 | Medium |
| 712 712 'Sales Related Occupations' | 712 | Medium |
| 713 713 'Sales Supervisors' | 713 | Medium |
| 721 721 'Customer Service Occupations' | 721 | Low |
| 722 722 'Customer Service Managers and Supervisors' | 722 | Low |
| 811 811 'Process Operatives' | 811 | Medium |
| 812 812 'Plant and Machine Operatives' | 812 | Low |
| 813 813 'Assemblers and Routine Operatives' | 813 | Low |
| 814 814 'Construction Operatives' | 814 | Low |
| 821 821 'Road Transport Drivers' | 821 | Medium |
| 822 822 'Mobile Machine Drivers and Operatives' | 822 | Low |
| 823 823 'Other Drivers and Transport Operatives' | 823 | Low |
| 911 911 'Elementary Agricultural Occupations' | 911 | Low |
| 912 912 'Elementary Construction Occupations' | 912 | Low |
| 913 913 'Elementary Process Plant Occupations' | 913 | Medium |
| 921 921 'Elementary Administration Occupations' | 921 | Medium |
| 923 923 'Elementary Cleaning Occupations' | 923 | Medium |
| 924 924 'Elementary Security Occupations' | 924 | Medium |
| 925 925 'Elementary Sales Occupations' | 925 | Medium |
| 926 926 'Elementary Storage Occupations' | 926 | Medium |
| 927 927 'Other Elementary Services Occupations' | 927 | Medium |

Table S3: Descriptive statistics by ethnicity, for all individuals aged 30+

|  |  | **Overall** | **White Scottish** | **White British or Irish** | **Other White** | **South Asian** | **African, Caribbean or Black** | **Other** |
| --- | --- | --- | --- | --- | --- | --- | --- | --- |
| Overall |  | **3060250, 100.0%** | **2624352, 85.8%** | **277226, 9.1%** | **78661, 2.6%** | **36696, 1.2%** | **11502, 0.4%** | **31813, 1.0%** |
| Age | 26-30 | 49830, 1.6% | 43753, 1.7% | 2942, 1.1% | 1391, 1.8% | 754, 2.1% | 213, 1.9% | 777, 2.4% |
|  | 31-35 | 256933, 8.4% | 219492, 8.4% | 16106, 5.8% | 10511, 13.4% | 4930, 13.4% | 1249, 10.9% | 4645, 14.6% |
|  | 36-40 | 263663, 8.6% | 213936, 8.2% | 19263, 6.9% | 17575, 22.3% | 6163, 16.8% | 2074, 18.0% | 4652, 14.6% |
|  | 41-45 | 253361, 8.3% | 204236, 7.8% | 22221, 8.0% | 13887, 17.7% | 5843, 15.9% | 2448, 21.3% | 4726, 14.9% |
|  | 46-50 | 300291, 9.8% | 250483, 9.5% | 28950, 10.4% | 9653, 12.3% | 4873, 13.3% | 1866, 16.2% | 4466, 14.0% |
|  | 51-55 | 343298, 11.2% | 294847, 11.2% | 32522, 11.7% | 7111, 9.0% | 3955, 10.8% | 1347, 11.7% | 3516, 11.1% |
|  | 56-60 | 343782, 11.2% | 298669, 11.4% | 32727, 11.8% | 5594, 7.1% | 2952, 8.0% | 935, 8.1% | 2905, 9.1% |
|  | 61-65 | 310349, 10.1% | 271549, 10.3% | 28923, 10.4% | 4446, 5.7% | 2612, 7.1% | 543, 4.7% | 2276, 7.2% |
|  | >65 | 938743, 30.7% | 827387, 31.5% | 93572, 33.8% | 8493, 10.8% | 4614, 12.6% | 827, 7.2% | 3850, 12.1% |
| Sex | Male | 1447656, 47.3% | 1235715, 47.1% | 135991, 49.1% | 35888, 45.6% | 19177, 52.3% | 5945, 51.7% | 14940, 47.0% |
|  | Female | 1612594, 52.7% | 1388637, 52.9% | 141235, 50.9% | 42773, 54.4% | 17519, 47.7% | 5557, 48.3% | 16873, 53.0% |
| Health board | Ayrshire and Arran | 227545, 7.4% | 208659, 8.0% | 14535, 5.2% | 2226, 2.8% | 765, 2.1% | 225, 2.0% | 1135, 3.6% |
|  | Borders | 66156, 2.2% | 52647, 2.0% | 11385, 4.1% | 1600, 2.0% | 139, 0.4% | 82, 0.7% | 303, 1.0% |
|  | Dumfries and Galloway | 91196, 3.0% | 73533, 2.8% | 15557, 5.6% | 1280, 1.6% | 251, 0.7% | 124, 1.1% | 451, 1.4% |
|  | Forth Valley | 165237, 5.4% | 146909, 5.6% | 13026, 4.7% | 3001, 3.8% | 1144, 3.1% | 258, 2.2% | 899, 2.8% |
|  | Grampian | 336417, 11.0% | 276060, 10.5% | 39851, 14.4% | 12247, 15.6% | 2472, 6.7% | 2028, 17.6% | 3759, 11.8% |
|  | Highland | 181845, 5.9% | 145008, 5.5% | 29528, 10.7% | 5324, 6.8% | 556, 1.5% | 281, 2.4% | 1148, 3.6% |
|  | Lothian | 471087, 15.4% | 378704, 14.4% | 53306, 19.2% | 22192, 28.2% | 6723, 18.3% | 2537, 22.1% | 7625, 24.0% |
|  | Orkney | 12641, 0.4% | * | * | * | * | * | * |
|  | Shetland | 13208, 0.4% | * | * | * | * | * | * |
|  | Western Isles | 15658, 0.5% | 13631, 0.5% | 1699, 0.6% | 202, 0.3% | 53, 0.1% | 12, 0.1% | 61, 0.2% |
|  | Fife | 209610, 6.8% | 183137, 7.0% | 19101, 6.9% | 4116, 5.2% | 1367, 3.7% | 424, 3.7% | 1465, 4.6% |
|  | Tayside | 234394, 7.7% | 201945, 7.7% | 21312, 7.7% | 6337, 8.1% | 2149, 5.9% | 579, 5.0% | 2072, 6.5% |
|  | Greater Glasgow and Clyde | 656602, 21.5% | 572464, 21.8% | 36810, 13.3% | 14821, 18.8% | 17574, 47.9% | 4249, 36.9% | 10684, 33.6% |
|  | Lanarkshire | 378654, 12.4% | 350757, 13.4% | 16850, 6.1% | 4863, 6.2% | 3453, 9.4% | 678, 5.9% | 2053, 6.5% |
| Occupation | Low risk | 1310187, 42.8% | 1133348, 43.2% | 127308, 45.9% | 27849, 35.4% | 9437, 25.7% | 3589, 31.2% | 8656, 27.2% |
|  | Med risk | 1093595, 35.7% | 944610, 36.0% | 80804, 29.1% | 36000, 45.8% | 15684, 42.7% | 3483, 30.3% | 13014, 40.9% |
|  | High risk | 573717, 18.7% | 481997, 18.4% | 64961, 23.4% | 11979, 15.2% | 5684, 15.5% | 3244, 28.2% | 5852, 18.4% |
|  | Unemployed | 82751, 2.7% | 64397, 2.5% | 4153, 1.5% | 2833, 3.6% | 5891, 16.1% | 1186, 10.3% | 4291, 13.5% |
| SIMD | 1 (most deprived) | 574758, 18.8% | 515901, 19.7% | 25259, 9.1% | 17835, 22.7% | 5808, 15.8% | 4043, 35.2% | 5912, 18.6% |
|  | 2 | 611792, 20.0% | 541963, 20.7% | 39292, 14.2% | 16248, 20.7% | 6714, 18.3% | 2218, 19.3% | 5357, 16.8% |
|  | 3 | 626504, 20.5% | 531940, 20.3% | 67159, 24.2% | 14472, 18.4% | 6035, 16.4% | 1659, 14.4% | 5239, 16.5% |
|  | 4 | 631337, 20.6% | 528833, 20.2% | 71684, 25.9% | 14302, 18.2% | 8385, 22.8% | 1715, 14.9% | 6418, 20.2% |
|  | 5 (least deprived) | 615859, 20.1% | 505715, 19.3% | 73832, 26.6% | 15804, 20.1% | 9754, 26.6% | 1867, 16.2% | 8887, 27.9% |
| Number of people in household | 1 | 500210, 16.3% | 443892, 16.9% | 40719, 14.7% | 8084, 10.3% | 2164, 5.9% | 1722, 15.0% | 3629, 11.4% |
|  | 2 | 1131781, 37.0% | 971962, 37.0% | 115887, 41.8% | 26588, 33.8% | 5705, 15.5% | 2820, 24.5% | 8819, 27.7% |
|  | 3 | 638293, 20.9% | 551804, 21.0% | 50802, 18.3% | 19575, 24.9% | 6327, 17.2% | 2495, 21.7% | 7290, 22.9% |
|  | 4 | 537715, 17.6% | 459147, 17.5% | 46493, 16.8% | 15233, 19.4% | 7907, 21.5% | 2290, 19.9% | 6645, 20.9% |
|  | 5+ | 252251, 8.2% | 197547, 7.5% | 23325, 8.4% | 9181, 11.7% | 14593, 39.8% | 2175, 18.9% | 5430, 17.1% |
| Education | Degree | 867678, 28.4% | 655001, 25.0% | 132658, 47.9% | 41505, 52.8% | 16181, 44.1% | 6395, 55.6% | 15938, 50.1% |
|  | No degree | 2192572, 71.6% | 1969351, 75.0% | 144568, 52.1% | 37156, 47.2% | 20515, 55.9% | 5107, 44.4% | 15875, 49.9% |
| Multigenerational household | No | 2934238, 95.9% | 2516065, 95.9% | 267067, 96.3% | 76872, 97.7% | 32704, 89.1% | 11234, 97.7% | 30296, 95.2% |
|  | Yes | 126012, 4.1% | 108287, 4.1% | 10159, 3.7% | 1789, 2.3% | 3992, 10.9% | 268, 2.3% | 1517, 4.8% |
| COVID-19 hosp or death | No | 3022476, 98.8% | 2590134, 98.7% | 274898, 99.2% | 78248, 99.5% | 36180, 98.6% | 11417, 99.3% | 31599, 99.3% |
|  | Yes | 37774, 1.2% | 34218, 1.3% | 2328, 0.8% | 413, 0.5% | 516, 1.4% | 85, 0.7% | 214, 0.7% |

Table S4: Descriptives of population aged 30-64, by ethnic groups.

|  | | **Overall** | **White Scottish** | **White British or Irish** | **Other White** | **South Asian** | **African, Caribbean or Black** | **Other** |
| --- | --- | --- | --- | --- | --- | --- | --- | --- |
| **Overall** | | **2064982, 100.0%** | **1747450, 84.6%** | **178271, 8.6%** | **69431, 3.4%** | **31644, 1.5%** | **10601, 0.5%** | **27585, 1.3%** |
| **Age** | **26-30** | 49830, 2.4% | 43753, 2.5% | 2942, 1.7% | 1391, 2.0% | 754, 2.4% | 213, 2.0% | 777, 2.8% |
|  | **31-35** | 256933, 12.4% | 219492, 12.6% | 16106, 9.0% | 10511, 15.1% | 4930, 15.6% | 1249, 11.8% | 4645, 16.8% |
|  | **36-40** | 263663, 12.8% | 213936, 12.2% | 19263, 10.8% | 17575, 25.3% | 6163, 19.5% | 2074, 19.6% | 4652, 16.9% |
|  | **41-45** | 253361, 12.3% | 204236, 11.7% | 22221, 12.5% | 13887, 20.0% | 5843, 18.5% | 2448, 23.1% | 4726, 17.1% |
|  | **46-50** | 300291, 14.5% | 250483, 14.3% | 28950, 16.2% | 9653, 13.9% | 4873, 15.4% | 1866, 17.6% | 4466, 16.2% |
|  | **51-55** | 343298, 16.6% | 294847, 16.9% | 32522, 18.2% | 7111, 10.2% | 3955, 12.5% | 1347, 12.7% | 3516, 12.7% |
|  | **56-60** | 343782, 16.6% | 298669, 17.1% | 32727, 18.4% | 5594, 8.1% | 2952, 9.3% | 935, 8.8% | 2905, 10.5% |
|  | **61-65** | 253824, 12.3% | 222034, 12.7% | 23540, 13.2% | 3709, 5.3% | 2174, 6.9% | 469, 4.4% | 1898, 6.9% |
| **Sex** | **Male** | 997243, 48.3% | 842408, 48.2% | 88225, 49.5% | 31844, 45.9% | 16424, 51.9% | 5507, 51.9% | 12835, 46.5% |
|  | **Female** | 1067739, 51.7% | 905042, 51.8% | 90046, 50.5% | 37587, 54.1% | 15220, 48.1% | 5094, 48.1% | 14750, 53.5% |
| **Health board** | **Ayrshire and Arran** | 147601, 7.1% | 135581, 7.8% | 8538, 4.8% | 1728, 2.5% | 637, 2.0% | 197, 1.9% | 920, 3.3% |
|  | **Borders** | 40777, 2.0% | 32737, 1.9% | 6276, 3.5% | 1329, 1.9% | 115, 0.4% | 70, 0.7% | 250, 0.9% |
|  | **Dumfries and Galloway** | 55830, 2.7% | 45993, 2.6% | 8136, 4.6% | 1015, 1.5% | 206, 0.7% | 108, 1.0% | 372, 1.3% |
|  | **Forth Valley** | 110996, 5.4% | 97912, 5.6% | 8511, 4.8% | 2588, 3.7% | 1006, 3.2% | 226, 2.1% | 753, 2.7% |
|  | **Grampian** | 229347, 11.1% | 184216, 10.5% | 26541, 14.9% | 11063, 15.9% | 2273, 7.2% | 1918, 18.1% | 3336, 12.1% |
|  | **Highland** | 114989, 5.6% | 92249, 5.3% | 16576, 9.3% | 4526, 6.5% | 458, 1.4% | 246, 2.3% | 934, 3.4% |
|  | **Lothian** | 328053, 15.9% | 253954, 14.5% | 38866, 21.8% | 20224, 29.1% | 5960, 18.8% | 2322, 21.9% | 6727, 24.4% |
|  | **Orkney** | 7909, 0.4% | * | * | * | * | * | * |
|  | **Shetland** | 8646, 0.4% | * | * | * | * | * | * |
|  | **Western Isles** | 9767, 0.5% | * | * | * | * | * | * |
|  | **Fife** | 137617, 6.7% | 119617, 6.8% | 11726, 6.6% | 3548, 5.1% | 1156, 3.7% | 376, 3.5% | 1194, 4.3% |
|  | **Tayside** | 150310, 7.3% | 127678, 7.3% | 13021, 7.3% | 5545, 8.0% | 1787, 5.6% | 529, 5.0% | 1750, 6.3% |
|  | **Greater Glasgow and Clyde** | 461636, 22.4% | 394679, 22.6% | 25427, 14.3% | 13134, 18.9% | 15001, 47.4% | 3965, 37.4% | 9430, 34.2% |
|  | **Lanarkshire** | 261504, 12.7% | 240900, 13.8% | 11090, 6.2% | 4194, 6.0% | 2963, 9.4% | 618, 5.8% | 1739, 6.3% |
| **Occupation** | **Low risk** | 904684, 43.8% | 775142, 44.4% | 85199, 47.8% | 24685, 35.6% | 8536, 27.0% | 3362, 31.7% | 7760, 28.1% |
|  | **Med risk** | 707573, 34.3% | 597964, 34.2% | 49517, 27.8% | 32577, 46.9% | 13353, 42.2% | 3229, 30.5% | 10933, 39.6% |
|  | **High risk** | 388299, 18.8% | 325120, 18.6% | 40610, 22.8% | 9721, 14.0% | 4947, 15.6% | 2886, 27.2% | 5015, 18.2% |
|  | **Unemployed** | 64426, 3.1% | 49224, 2.8% | 2945, 1.7% | 2448, 3.5% | 4808, 15.2% | 1124, 10.6% | 3877, 14.1% |
| **SIMD** | **1 (most deprived)** | 408541, 19.8% | 360518, 20.6% | 17282, 9.7% | 16494, 23.8% | 5148, 16.3% | 3828, 36.1% | 5271, 19.1% |
|  | **2** | 415110, 20.1% | 362128, 20.7% | 25539, 14.3% | 14802, 21.3% | 5895, 18.6% | 2049, 19.3% | 4697, 17.0% |
|  | **3** | 413810, 20.0% | 348900, 20.0% | 41096, 23.1% | 12580, 18.1% | 5226, 16.5% | 1501, 14.2% | 4507, 16.3% |
|  | **4** | 422400, 20.5% | 350143, 20.0% | 45566, 25.6% | 12364, 17.8% | 7242, 22.9% | 1560, 14.7% | 5525, 20.0% |
|  | **5 (least deprived)** | 405121, 19.6% | 325761, 18.6% | 48788, 27.4% | 13191, 19.0% | 8133, 25.7% | 1663, 15.7% | 7585, 27.5% |
| **Number of people in household** | **1** | 260852, 12.6% | 228147, 13.1% | 20839, 11.7% | 5940, 8.6% | 1582, 5.0% | 1490, 14.1% | 2854, 10.3% |
|  | **2** | 555643, 26.9% | 465469, 26.6% | 54607, 30.6% | 21891, 31.5% | 4276, 13.5% | 2458, 23.2% | 6942, 25.2% |
|  | **3** | 517165, 25.0% | 445280, 25.5% | 39274, 22.0% | 18243, 26.3% | 5528, 17.5% | 2342, 22.1% | 6498, 23.6% |
|  | **4** | 500229, 24.2% | 427298, 24.5% | 42536, 23.9% | 14659, 21.1% | 7289, 23.0% | 2217, 20.9% | 6230, 22.6% |
|  | **5+** | 231093, 11.2% | 181256, 10.4% | 21015, 11.8% | 8698, 12.5% | 12969, 41.0% | 2094, 19.8% | 5061, 18.3% |
| **Education** | **Degree** | 633630, 30.7% | 470524, 26.9% | 91158, 51.1% | 36917, 53.2% | 14689, 46.4% | 5943, 56.1% | 14399, 52.2% |
|  | **No degree** | 1431352, 69.3% | 1276926, 73.1% | 87113, 48.9% | 32514, 46.8% | 16955, 53.6% | 4658, 43.9% | 13186, 47.8% |
| **Multigenerational household** | **No** | 1999963, 96.9% | 1691335, 96.8% | 174207, 97.7% | 68367, 98.5% | 29041, 91.8% | 10425, 98.3% | 26588, 96.4% |
|  | **Yes** | 65019, 3.1% | 56115, 3.2% | 4064, 2.3% | 1064, 1.5% | 2603, 8.2% | 176, 1.7% | 997, 3.6% |
| **COVID-19 hosp or death** | **No** | 2053927, 99.5% | 1737709, 99.4% | 177674, 99.7% | 69197, 99.7% | 31356, 99.1% | 10534, 99.4% | 27457, 99.5% |
|  | **Yes** | 11055, 0.5% | 9741, 0.6% | 597, 0.3% | 234, 0.3% | 288, 0.9% | 67, 0.6% | 128, 0.5% |

Table S5: Summary of inverse propensity weights for disaggregated ethnicity

|  | **Minimum** | **1st quartile** | **Median** | **Mean** | **3rd quartile** | **Maximum** |
| --- | --- | --- | --- | --- | --- | --- |
| **Exposure** | | | | | | |
| Ethnicity | 0.35 | 0.94 | 0.98 | 0.99 | 1.05 | 1.95 |
| Ethnicity (population aged 30+) | 0.35 | 0.98 | 0.99 | 1.04 | 2.05 | 0.98 |
| **Mediators** | | | | | | |
| SIMD (5 groups) | 0.56 | 0.82 | 0.96 | 1.00 | 1.10 | 1.83 |
| Education (binary - population aged 30+) | 0.60 | 0.92 | 1.04 | 1.09 | 1.21 | 2.08 |
| Household size (5 groups) | 0.35 | 0.76 | 0.82 | 0.98 | 1.06 | 2.89 |
| Multigenerational household (binary) | 0.64 | 0.99 | 0.99 | 1.00 | 1.01 | 1.30 |
| **Combined ethnicity and mediator weights** |  | | | | | |
| SIMD (5 groups) | 0.20 | 0.79 | 0.95 | 0.99 | 1.12 | 3.58 |
| Education (binary - population aged 30+) | 0.24 | 0.88 | 1.02 | 1.07 | 1.21 | 4.26 |
| Household size (5 groups) | 0.16 | 0.72 | 0.82 | 0.97 | 1.09 | 5.64 |
| Multigenerational household (binary) | 0.22 | 0.94 | 0.98 | 0.99 | 1.05 | 2.54 |

Table S6: Total effect and counterfactual disparity measures and proportional eliminated (PE) in severe COVID-19 on aggregated ethnicity.

|  |  | HR (95% CI) | PE (95% CI) |
| --- | --- | --- | --- |
| **Total effect** | | | |
| Ethnicity | White | 1 |  |
|  | Non-White | 1.37 (1.28, 1.47) | n/a |
| **Counterfactual disparity measures, after accounting for:** | | | |
| SIMD (5 groups) | White | 1 |  |
|  | Non-White | 1.19 (1.01, 1.40) | 52.73% (52.57%, 52.89%) |
| Education (binary - population aged 30+) | White | 1 |  |
|  | Non-White | 1.74 (1.52, 1.99) | -98.81% (-99.06%, -98.75%) |
| Household size (5 groups) | White | 1 |  |
|  | Non-White | 1.04 (0.87, 1.23) | 88.58% (88.42%, 88.75%) |
| Multigenerational household (binary) | White | 1 |  |
|  | Non-White | 1.38 (1.28, 1.49) | -3.20% (-3.24%, -3.15%) |

Table S7: Summary of weights aggregated ethnicity.

|  | **Minimum** | **1st quartile** | **Median** | **Mean** | **3rd quartile** | **Max.** |
| --- | --- | --- | --- | --- | --- | --- |
| **Exposure** | | | | | | |
| Ethnicity | 0.5 | 0.98 | 0.99 | 0.99 | 1.01 | 1.08 |
| Ethnicity (population aged 30+) | 0.5 | 0.98 | 0.99 | 0.99 | 1.01 | 1.07 |
| **Mediators** | | | | | | |
| SIMD (5 groups) | 0.56 | 0.82 | 0.96 | 1 | 1.11 | 1.86 |
| Education (binary - population aged 30+) | 0.62 | 0.9 | 0.97 | 1 | 1.07 | 1.62 |
| Household size (5 groups) | 0.35 | 0.76 | 0.82 | 0.98 | 1.06 | 2.88 |
| Multigenerational household (binary) | 0.64 | 0.99 | 0.99 | 1 | 1.01 | 1.3 |
| **Combined ethnicity and mediator weights** | | | | | | |
| SIMD (5 groups) | 0.3 | 0.82 | 0.95 | 0.99 | 1.09 | 2 |
| Education (binary - population aged 30+) | 0.33 | 0.89 | 0.97 | 0.99 | 1.08 | 1.74 |
| Household size (5 groups) | 0.22 | 0.75 | 0.82 | 0.97 | 1.06 | 3.1 |
| Multigenerational household (binary) | 0.32 | 0.98 | 0.99 | 0.99 | 1.01 | 1.4 |
